# Embracing the sustainable horizons through bioenergy innovations: a path to a sustainable energy future

**DOI:** 10.3389/fchem.2024.1416102

**Published:** 2024-07-30

**Authors:** Rubén Blay-Roger, Maria Saif, Luis F. Bobadilla, Tomas Ramirez-Reina, Muhammad Asif Nawaz, José Antonio Odriozola

**Affiliations:** Department of Inorganic Chemistry and Materials Sciences Institute, University of Seville-CSIC, Seville, Spain

**Keywords:** bio energy, sustainability, resource management, biofuel, policymaking

## Abstract

The urgent need for mitigating climate change necessitates a transformative shift in energy production and consumption paradigms. Amidst this challenge, bioenergy emerges as a pivotal contributor to the global energy transition, offering a diverse array of solid, liquid, and gaseous fuels derived from biomass. This mini review delves into the unique potential of bioenergy innovations, particularly renewable diesel, bio jet fuel, and ethanol, to reduce greenhouse gas emissions and transform various industries. The article highlights critical technological advancements, supportive policies, and cross-sector collaboration essential for a sustainable energy transition. Specific challenges such as ensuring a consistent biomass feedstock supply, decentralizing processing units, and navigating complex regulatory frameworks are examined. Innovative solutions like decentralized biomass processing and enhanced biomass logistics are discussed as pathways to overcome these barriers. The review provides specific recommendations for near-term policies and strategies to support decentralized facilities, showcasing bioenergy’s role in achieving a sustainable future.

## Graphical Abstract

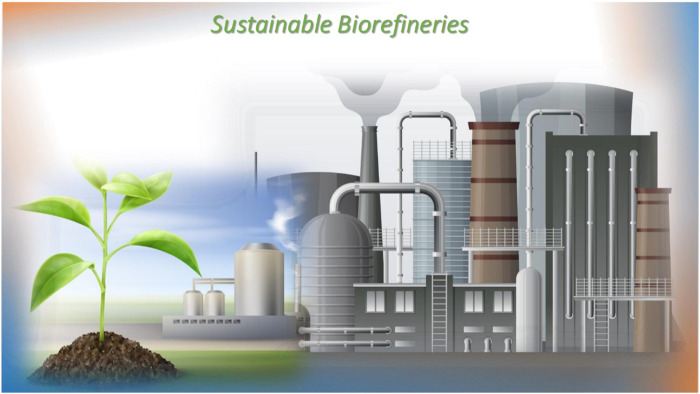

## Biofuel innovations

The pressing challenge of climate change demands a radical transformation in how we produce and consume energy. In this context, bioenergy emerges as an interesting option in the global energy transition, derived from biomass to a diverse array of solid, liquid, and gaseous fuels. Intermediate products like C_5_ and C_6_ sugars, syngas, lignin, and pyrolytic liquids serve as platforms for further processing into energy carriers, chemicals, materials, and food/feed components (Ubando et al., 2021; Gholizadeh et al., 2023a; Bachs-Herrera et al., 2023; Blasi et al., 2023). While the promise of bioenergy innovations for renewable diesel, bio jet fuel, and ethanol, etc., mitigating greenhouse gas (GHG) emissions and improving air quality could extend beyond environmental benefits. In the quest for sustainable chemistry, researchers are exploring bio-based building blocks across various carbon chains, holding promise for revolutionizing industries from pharmaceuticals to polymer (Nawaz et al., 2022; Saif et al., 2022; Dutta et al., 2023; Emran et al., 2023). As we journey through the intricate pathways of bio-based building blocks, it becomes evident that sustainable chemistry holds the key to unlocking a greener, more resilient future. This transition is not just an environmental imperative but also a commercial opportunity, with significant investments flowing into second-generation biofuels sourced from non-food biomass materials (Chein et al., 2021; Nawaz et al., 2023). The projected growth of biofuels is robust, driven by technological advancements, environmental policies, and evolving market dynamics. However, realizing the full potential of bioenergy requires overcoming substantial challenges. Ensuring a consistent and diverse supply of biomass feedstocks is crucial, necessitating innovation in agricultural practices and feedstock logistics (Andersen et al., 2016; Ooi et al., 2019; Sun et al., 2019; Pham et al., 2020; Chein et al., 2021; Calvo-Flores et al., 2022; de Souza Vandenberghe et al., 2022; Gholizadeh et al., 2023b; Hu et al., 2023; Nawaz et al., 2023; González-Arias et al., 2024; Zhao et al., 2024). Moreover, supportive policy frameworks are essential to foster technological innovation and ensure the economic viability of biofuels. Additionally, initiatives such as research and development funding, infrastructure development grants, and sustainability certification programs further support the growth of biofuels by addressing technological barriers and ensuring environmental sustainability (Paes et al., 2022). This article delves into these aspects, exploring the transformative potential of bioenergy and the bioeconomy, highlighting the importance of sustainable chemistry in reshaping our energy and materials landscape, while advocating for continuous research and development to unlock new opportunities.

## Synthetic fuels scenario

**FIGURE 1:**
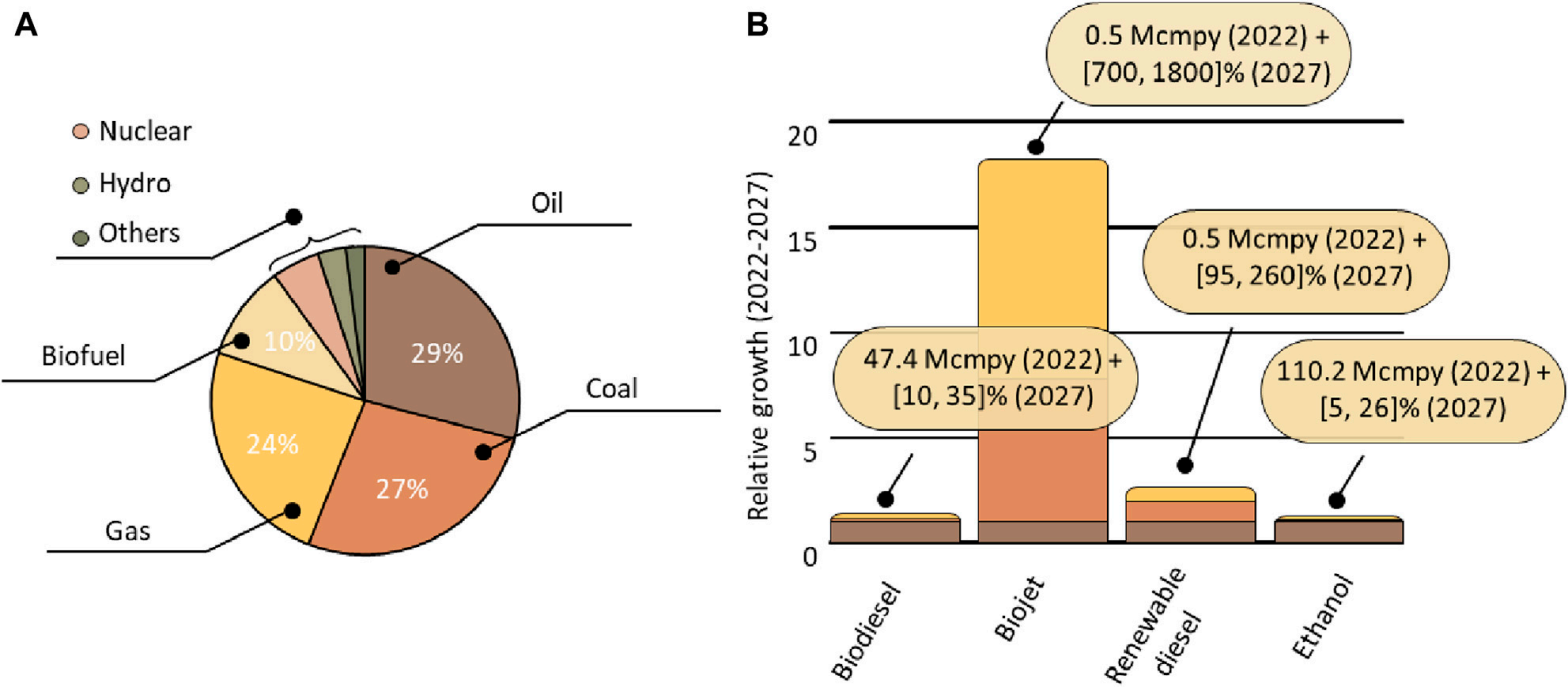
Synthetic fuels scenarios, **(A)** Current sources of energy consumption. **(B)** Expected growth of bioresources. note: Mcmpy (Million cubic meters per year).

**FIGURE 2:**
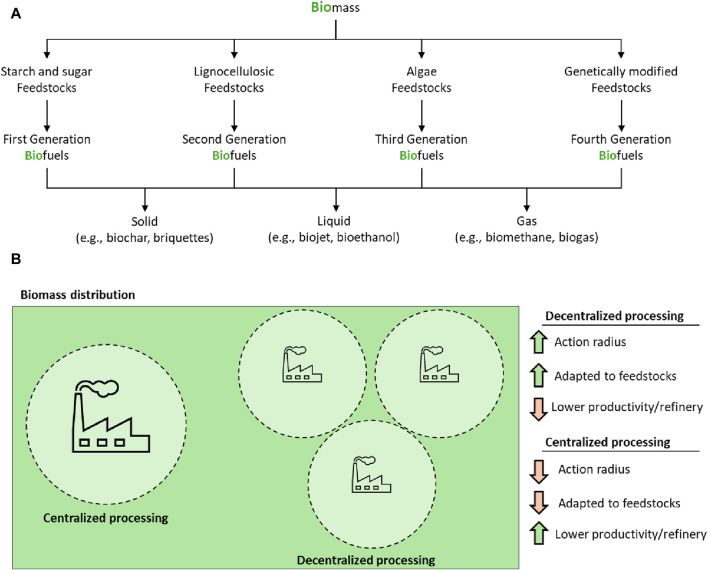
**(A)** General scheme for biofuels production. **(B)** General scheme for biomass processing.

The countdown to the end of the largest energy vector used to date has begun. With a total estimate of 1.6 billion barrels of oil and a consumption rate of 3 million barrels per day (assuming the current consumption rate), we have a margin of 47 years to find alternatives. The ongoing energy transition requires us to undertake the challenging task of decentralizing energy production, consumption, and utilization. However, this task is not simple, as over 3/4 of global energy production is provided using fossil resources (Figure 1A). The main alternative is the use of biofuels, which currently provide 10% of the energy but have immense potential to take up a significant portion of the fossil fuel’s mantle. Biofuels are a term used to refer to all hydrocarbons produced from biomass (Figure 2A). Their commercial interest lies in the transformation of low-value and low-energy-density waste (such as wood, agricultural residues, waste, algae, and plant matter) into high-energy-density and value-added products (Rodriguez et al., 2021; Xavier et al., 2022). The demand for biofuels is continuously rising year by year, with a promising long-term projection. In 2022 the combined production of the main biofuels (biodiesel, bio jet, renewable diesel, and ethanol) stood at 171 Mmc (million cubic meters). Currently, biodiesel production is estimated at 47 Mcmpy (million cubic meters per year), and it is expected to experience a further increase of 10%–35% by 2027 (Figure 1B) (Duan et al., 2022; Pérez-Almada et al., 2023). The outlook for other biofuels, such as renewable diesel, bio jet, and ethanol, is also positive, with robust growth anticipated in the coming years (Figure 1B).

### Decentralization of biomass processing units

Despite the estimated growth, the biofuel industry faces three major challenges in the energy transition: transportation, supply, and raw material. Historically, refining plants have been built close to vast oil reservoirs to ensure an uninterrupted supply of raw materials. Artificial fuel synthesis plants (e.g., coal to liquid-CTL, gas to liquid-GTL) have also adopted these measures to prevent production disruptions. New biorefineries have followed this trend, constructing plants near biomass reservoirs to minimize the transportation costs of raw materials. However, the low energy density stored in biomass (0.08 W/m^2^) compared to the high density of fossil resources like natural gas (482 W/m^2^) highlights the significant amount of biomass that must be transported to achieve energy comparable to gas. This limits the collection and transport radius of biorefineries to a few tens of kilometers around the facility to ensure economic viability. On the other hand, the seasonal dependence of biomass production limits continuity in production, alternating months of high biomass availability with months of low availability. This generates instability in processing plants, causing fluctuations in the production chain. Finally, the origin of the raw material is another handicap. The chemical nature of the raw material often conditions the design of a processing plant, making it difficult to use a single facility for different biomass sources. These factors lead to the design of biorefineries at specific points on the map where a high density of biomass near the processing plant can be combined with a continuous supply of raw materials. This strategy, inspired by models based on the utilization of oil, limits the energy potential of biomass, with trillions of joules lost in the natural catabolic processes of biomass. Jagtar Singh and co-workers studied how the problem of transporting biomass to processing plants is a bottleneck for unleashing the potential of biomass. By studying only, the utilization of agricultural biomass in a region of Punjab (India), they found that a total of 900 MW was wasted, which could be transformed into a loss of 20 Kg of diesel per second. These data highlight a reality: The utilization of biomass is underexploited. If we genuinely want to harness the latent potential of biomass, it is necessary to fully address the three bottlenecks mentioned. An interesting solution is to bet on the decentralization of biomass utilization through the downscaling of biorefineries.

The decentralization of biomass processing to produce biofuels is an intriguing option, with one of the most significant benefits being the ability to access a wider range of biomass niches (Figure 2B). Large processing plants are constrained by transportation logistics, as it is only economically feasible to transport biomass from relatively nearby locations. For every additional kilometer, the biomass is transported, the added value of the resulting biofuel decreases. However, smaller, and mobile plants can relocate to various locations, utilizing local resources that would otherwise be inaccessible or not economically viable. This not only increases the total quantity of biomass available but also encourages the use of agricultural, forestry, and urban waste that would otherwise be wasted. Specialization is another crucial aspect that justifies decentralization. Each type of biomass has unique characteristics that require specific conversion processes to maximize efficiency and minimize environmental impact. A conventional processing plant has a limited capacity to adapt to the raw material to be processed. This explains why there are large masses of biomass waste within the radius of action of a biorefinery that are wasted in natural decomposition processes. In practical terms, biomass whose composition is tolerable within the limitations of the processing plant is processed. Small, mobile processing plants can be designed to suit a specific type of biomass, adjusting their methods and technologies to optimize performance. For example, one plant might specialize in lignocellulosic biomass, while another might be optimized for agricultural waste. This specialization not only improves processing efficiency but also ensures that each type of biomass is converted in the most sustainable and environmentally friendly way possible. Decentralization also has a positive impact on the overall sustainability of the biofuel production process. By reducing the need to transport biomass over long distances, the carbon footprint associated with the process is significantly reduced. Furthermore, by using local biomass waste, waste management is improved, and circular economy is promoted, where waste becomes valuable resources. However, although promising, pursuing this approach entails several significant challenges. These challenges range from the de-escalation of processing plants to the integration of renewable energy sources, including the need to simplify the operation of these facilities. The transition from large, centralized biomass processing plants to smaller, mobile units involves not only a change in the physical scale of the facilities, but also the reengineering of processes to maintain efficiency at a reduced scale. Existing technologies in large plants may not be directly transferable to a smaller scale, requiring significant innovation in equipment design and operation. The implementation of microreactors for small-scale catalytic processing represents another challenge. These systems must be efficient, capable of handling variations in biomass quality and type, and robust enough to operate in different environments. The design of microreactors that meet these requirements is an emerging field and will require considerable technological development. The integration of renewable energy sources, such as solar panels, is essential to guarantee the sustainability of decentralized plants. However, this presents challenges in terms of ensuring a constant and reliable source of energy, given the inherent variations in the availability of resources such as sunlight. The combination of different renewable energy sources and energy storage are key areas that need development. Another major challenge is ensuring that decentralized plants are simple enough to be operated by a single operator. This involves not only an intuitive and easy-to-use design, but also the incorporation of automation and remote-control systems. Operator training and skills are also crucial aspects to consider. Additionally, the maintenance and logistics of multiple decentralized plants presents a challenge. Each unit must be maintained efficiently to ensure its optimal operation, which requires a well-organized logistics network and possibly a remote monitoring system to quickly identify and resolve problems.

### Feedstock availability and diversity

Ensuring a consistent and diverse supply of feedstock is crucial for the widespread adoption of biofuels as a sustainable energy source. Addressing challenges related to land availability, competition with food crops, and feedstock quality requires innovation, investment, and collaborative efforts across various sectors (Pinheiro Pires et al., 2019; Pérez-Almada et al., 2023). By implementing sustainable practices and leveraging technological advancements, biofuel producers can overcome these barriers and contribute to a more sustainable energy future. One of the primary technological barriers in biofuel generation lies in ensuring a consistent and diverse supply of feedstock (Rathour et al., 2023). Ensuring a continuous supply of processable residues to such plants requires the use of substantial amounts of energy for transportation to processing points. This often represents a handicap that is not worth it from an economic and environmental point of view. The amount of biomass on earth is estimated at 550 Gt of carbon, of which 320 is biomass available on the surface (Aguilar-Rivera and Olvera-Vargas, 2022). This amount of biomass is continuously recirculated through the natural carbon cycle, responsible for processing, transforming, and recirculating the forms in which the organic matter is found. Much of this complex organic matter is degraded through metabolic cycles of natural decomposition to produce CO_2_ which will eventually be fixed again by photosynthetic organisms to produce complex molecules (Alibardi et al., 2020). The availability of suitable biomass feedstocks, such as agricultural residues, forestry waste, and energy crops, is crucial for sustainable biofuel production. However, several challenges must be addressed to overcome these obstacles. Limited land availability presents a significant challenge in sourcing biomass feedstocks for biofuel production. Competition with food crops for arable land further complicates the issue, raising concerns about food security and land-use conflicts. Additionally, variations in feedstock quality and composition pose challenges in optimizing biofuel production processes and ensuring consistent fuel quality. Securing a consistent supply of sustainable biomass feedstocks poses a fundamental challenge for biofuel producers. Balancing the utilization of agricultural and forestry residues with preserving soil health and biodiversity is paramount. Over-reliance on specific feedstocks or unsustainable harvesting practices can lead to deforestation, soil degradation, and adverse environmental impacts. Addressing these challenges requires a multi-faceted approach. Advancements in biomass cultivation techniques, such as precision agriculture and agroforestry, can increase biomass yields while minimizing environmental impacts. Genetic engineering and biotechnology offer opportunities to develop energy crops with improved traits, such as higher yields, stress tolerance, and enhanced conversion efficiency. Furthermore, implementing sustainable land management practices and promoting crop rotation can help maintain soil health and biodiversity while supporting biofuel feedstock production.

## Technological advancements, resource efficiency and future recommendations

Modern bioenergy, powered by efficient biomass-based fuels, is instrumental in providing cleaner energy and improving health outcomes while combating climate change (Galbe and Wallberg, 2019; Gulshan Kumar et al., 2022; Gholizadeh et al., 2023a; Bulgari et al., 2023; Pathak et al., 2023; Paladino and Neviani, 2024). Despite its significant benefits, the sustainable implementation of bioenergy remains limited. Realizing its full potential requires collaborative efforts of environmental imperatives, supportive policy frameworks, cross-sector collaboration, and dynamic market conditions to unlock new opportunities and drive the future of biofuels.

**FIGURE 3:**
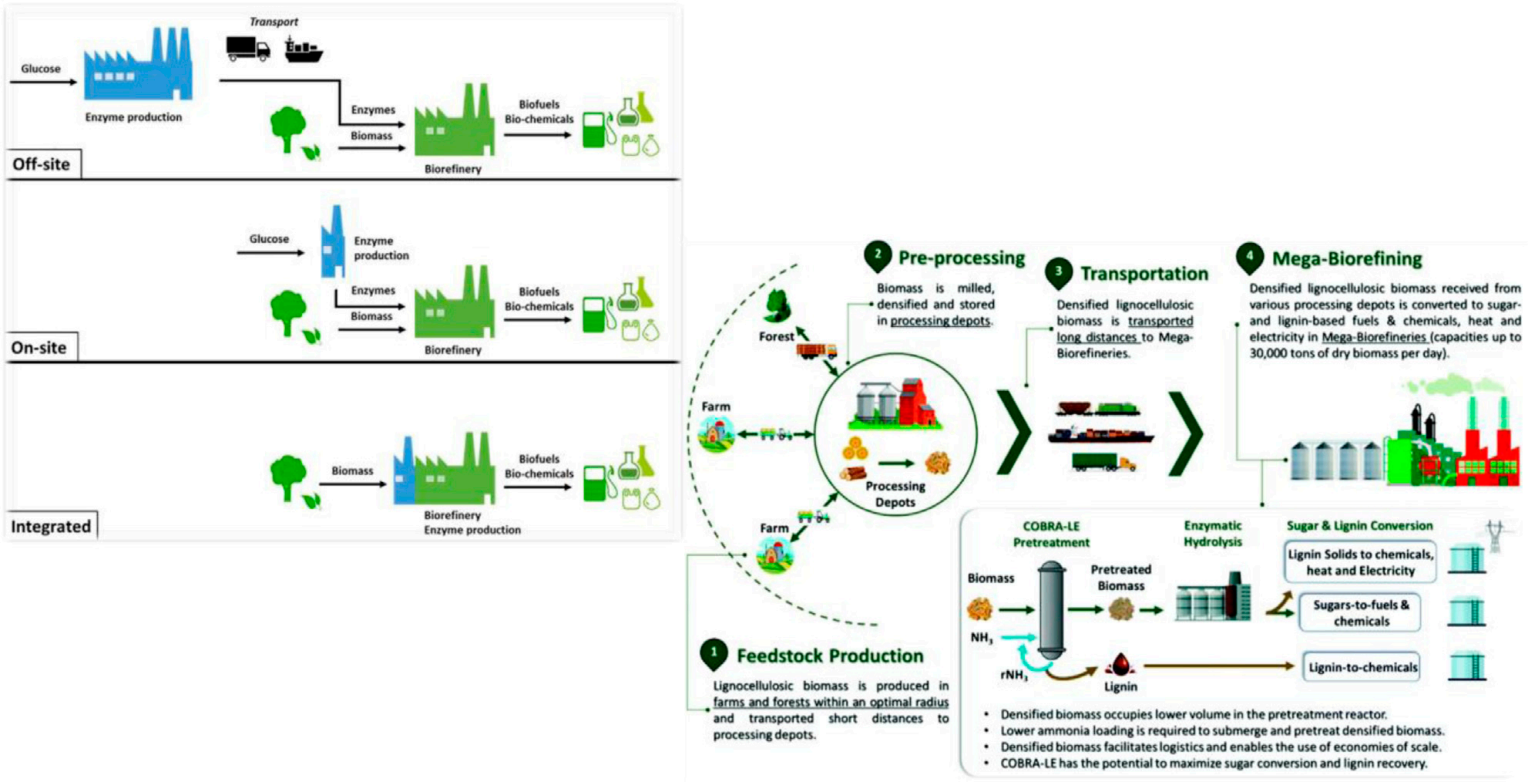
Centralization, Resource management and technical advancement in biorefineries by feedstock processing and innovations, Reproduced with permission from ref (Morais et al., 2022). Copyright 2022 Royal Society of Chemistry, and ref (Dragone et al., 2020). Copyright 2020 E.lsevier.

Technological advancements are the cornerstone of biofuel growth, enabling more efficient and cost-effective production processes. Improved methods for biomass conversion and biofuel synthesis significantly enhance the viability and scalability of biofuel production. Such as decentralizing biomass processing through smaller, mobile plants offers access to diverse biomass sources that allows for the efficient processing of varied feedstocks. Technologies such as combustion, anaerobic digestion, and pyrolysis facilitate diverse applications from electricity generation to industrial heat. The complexity of bioenergy, with its diverse feedstocks and technologies, necessitates robust governance for sustainable sourcing and efficient conversion. Sustainable biomass production is integral to transitioning to a circular economy by replacing fossil fuels with renewable resources. Emerging technologies like algal biofuels, which promise higher yields and lower resource requirements, highlight the dynamic landscape of biofuel research. Exploring diverse bioenergy pathways and advanced conversion technologies reveals the potential of agricultural residues, waste streams, and non-food crops for renewable energy production. Biomass’s complexity and diversity make it suitable for producing energy, fibers, proteins, and base chemicals. However, optimizing feedstocks, processes, and product combinations remains challenging. Advances in bioengineering can enhance energy crops and microorganisms, leading to better biomass yields. Integrated biorefineries optimize biomass utilization to produce a range of valuable products, from bioethanol to renewable chemicals. Various conversion pathways, including biochemical, thermochemical, and hybrid processes, offer different advantages and trade-offs in terms of feedstock flexibility, product yield, and process efficiency (Zhou et al., 2023; Badoga et al., 2024). Innovations in reactor design, catalyst development, process integration, and optimization are essential for enhancing biofuel production’s viability and sustainability (Agun et al., 2022). Efficient biomass-to-biofuel conversion remains a technological challenge, particularly regarding scalability, energy efficiency, and cost-effectiveness (Saravanan et al., 2023; Talero and Kansha, 2023). Resource efficiency and waste management are critical for sustainable biofuel generation, aiming to minimize resource inputs, waste generation, and environmental pollution throughout the production process (Figure 3). Efficient utilization of biomass feedstocks, waste valorization, and co-product utilization can enhance resource efficiency and reduce environmental burdens. Innovative technologies, such as biorefinery concepts, integrated production systems, and circular economy approaches, offer opportunities to optimize resource use and minimize environmental impacts in biofuel generation. Ensuring the environmental sustainability of biofuel generation involves comprehensive lifecycle assessments (LCAs) to evaluate the environmental impacts from cradle to grave. Advances in LCA methodologies, data availability, and modeling tools are needed to improve the accuracy and reliability of sustainability assessments and inform decision-making.

Economic constraints, such as high production costs, low market demand for renewable alternatives, and competition from biofuel markets, hinder the deployment of bio-based chemicals and materials (Widianingsih et al., 2021). To address these barriers, a comprehensive approach is necessary. Continued support for pilot and demonstration plants is essential. Data from these plants can inform early-stage techno-economic analyses, optimize processes, and reduce costs. Utilizing shared facilities, clear project classification, and increased equity investments can help lower scaling-up costs. Funding biomass resource assessments, supporting supply chain projects, and establishing supply companies are crucial for a robust biomass resource base (Topcu and Turgut, 2023). Aligning material classification across member states and implementing global matchmaking programs can streamline biomass resource development. Mandates for bio-based products and lower lifecycle GHG emissions, along with public procurement standards for low GHG products, can drive market demand. Addressing cost competitiveness through taxation and varied policy support measures will enhance market viability. Focusing on non-crop routes with low land use impact and continued public co-financing for pilot plants is necessary for scaling to commercial levels. Validating techno-economic models with pilot plant data, supporting shared facilities, and increasing equity investments will foster growth in the biorefinery sector. Supportive policy frameworks are essential to encourage technological innovation and ensure the economic viability of biofuels. Specific examples of successful policies include the Renewable Fuel Standard (RFS) in the United States, which mandates a certain volume of renewable fuel to replace or reduce the quantity of petroleum-based transportation fuel, heating oil, or jet fuel​​. The European Union’s Renewable Energy Directive (RED) sets targets for the share of energy from renewable sources, including biofuels, in the transport sector (Scarlat et al., 2018). These policies have strengths such as driving market demand and encouraging investment in biofuel technologies, but they also have weaknesses like market volatility and implementation challenges. Additionally, initiatives such as research and development funding, infrastructure development grants, and sustainability certification programs further support the growth of biofuels by addressing technological barriers and ensuring environmental sustainability. For instance, the Biomass Crop Assistance Program (BCAP) in the U.S. provides financial assistance to owners and operators of agricultural and non-industrial private forest land who wish to establish, produce, and deliver biomass feedstocks (Epplin et al., 2007; Coyle, 2010). Evaluating the effectiveness of these incentives is crucial. For example, BCAP has shown success in increasing biomass availability but has faced challenges related to funding consistency and participant enrollment. Therefore, these policies for renewable fuel standards or carbon pricing mechanisms create market demand for biofuels and incentivize investment in renewable energy technologies, thus encouraging localized biomass utilization, reducing transportation emissions, and enhancing rural economic development.
